# Influences of Pre-fracture Mobility and Early Mobility on Healthcare Outcome Measures in Older Patients Undergoing Hip Fracture Surgery

**DOI:** 10.1007/s00223-025-01475-6

**Published:** 2026-01-23

**Authors:** Radcliffe Lisk, Keefai Yeong, Hazel Watters, Christopher H. Fry, Thang S. Han

**Affiliations:** 1 Department of Orthogeriatrics, Ashford and St Peter’s NHS Foundation Trust, Guildford Road, Chertsey, Surrey, KT16 0PZ UK; 2 Department of Orthopaedic Trauma, Ashford and St. Peter’s NHS Foundation Trust, Guildford Road, Chertsey, Surrey, KT16 0PZ UK; 3 School of Physiology, Pharmacology and Neuroscience, University of Bristol, Bristol, BS8 1TD UK; 4 Institute of Cardiovascular Research, Royal Holloway, University of London, Egham, Surrey, TW20 0EX UK; 5 Department of Endocrinology, Ashford and St Peter’s NHS Foundation Trust, Guildford Road, Chertsey, Surrey, KT16 0PZ UK

**Keywords:** Hip fractures, Pre-and post-surgical mobility, Health outcomes, Discharge planning, Key performance indicators

## Abstract

Early mobilisation (within 1-day of hip fracture surgery) improves health-related outcomes, However, the role of pre-fracture mobility has hitherto been overlooked. We postulated that both pre-fracture and early mobility independently influence patient outcome measures when admitted with hip fractures. A total of 3134 patients (71.2% women) aged 60–107 year undergoing hip fracture surgery between 01-April-2014 and 03-June-2022 were studied. Pre-fracture mobility and early mobility were regressed simultaneously in multivariable logistic models to predict outcomes and discharge destinations. Data are presented as age- and sex-adjusted odds ratios (OR) and 95% confidence intervals (CI). Compared to patients able to mobilise freely before a fracture, those who could only mobilise outdoors with two aids/frame or were limited to indoors had: a greater risk of death, OR = 1.87 (1.20–2.91); hospital stay ≥ 16days (upper quartile), OR = 1.65 (1.33–2.05); or pressure ulcers, OR = 1.91 (1.00–3.68). Also, they were more likely to require rehabilitation, OR = 1.99 (1.60–2.48) and residential/nursing care, OR = 2.26 (1.40–3.64) and less likely to return home; OR = 0.43 (0.35–0.53). Compared to patients who mobilised early within 1-day of hip fracture surgery, those unable to mobilise early had greater risk of death, OR = 4.17 (2.47–7.05); hospital stay ≥ 16days, OR = 2.05 (1.55–2.72); pressure ulcers, OR = 3.31 (1.33–8.26), and more likely to require residential/nursing care, OR = 2.95 (1.40–6.22) and less likely to return home, OR = 0.58 (0.43–0.77). Pre-fracture mobility and early mobility are independent indicators of health outcomes and level of care. Thus, both should be considered by multidisciplinary teams reviewing management and discharge planning for elderly adults undergoing hip fracture surgery.

**Supplementary Information:**

The online version contains supplementary material available at 10.1007/s00223-025-01475-6.

## Introduction

Global population ageing has been gaining pace over the past several decades [1]. In 2021, the total population in England and Wales was almost 60 million, of whom 18.6% (11.1 million) were ≥ 65 years, and 0.9% (527,900) ≥ 90 years [2]. The ageing process leads to a progressive functional decline in the neuro-musculoskeletal system, and encompasses sarcopenia, bone fragility and neuromuscular dysfunction. This decline is clinically manifest as cognitive impairment, frailty, impaired movement and coordination [3–7]. The rate of this decline varies between individuals and may be accelerated by factors such as underlying chronic diseases, malnutrition, drugs and immobility. Ultimately, age-related neuro-musculoskeletal decline predisposes older individuals to frequent falls and bone, in particular hip, fractures.

Because more individuals are living to an older age, an increase of hospital admissions for hip fractures has emerged [8], resulting in a current need for greater provision of care and resources [9, 10]. Disability due to hip fracture is common and requires high levels of care both in hospital and the community once discharged from hospital [11–14]. To improve care for such patients, research has focussed on identifying risk factors of outcome measures, primarily the ability to recover bodily function in a timely fashion. Previous studies have demonstrated that early mobilisation after hip fracture surgery is associated with improved outcome measures [15–19]. Pre-fracture mobility also relates closely to outcome measures amongst patients with hip fractures [20, 21]. However, the role of pre-fracture mobility has consistently been overlooked in analyses of early mobility and associated healthcare outcomes. Therefore, this limits the ability to identify patients with potential for remobilisation, with consequent delayed management and discharge planning. In this study, we postulate that pre-fracture mobility and early mobility (within 1-day of hip fracture surgery) both have independent influences on healthcare outcomes and discharge destinations.

## Methods

### Study Design, Participants and Setting

In this cross-sectional study, 3134 older (≥ 60 years) patients consecutively admitted for hip fractures and were operated between 1st April 2014 and 3rd June 2022 at a single hospital (Ashford and St Peter’s Hospitals NHS Foundation Trust, Surrey, UK) were analysed.

### Data Collection

Clinical data were collected through our participation in the National Hip Fracture Database (NHFD) [22, 23], co-ordinated by an orthopaedic trauma nurse and orthogeriatric consultant. Data included demographic information (age, sex and residence), as well as pre-fracture mobility level, in-hospital mobility level on day of, or one day after, surgery (assessed by physiotherapists); mortality; pressure ulcers; length of stay (LOS); discharge destinations; American Society of Anesthesiologists (ASA) score, type of hip fracture, side of hip fracture, delay to surgery, and Abbreviated Mental Test scores (AMTS). Nutritional status was assessed using the Malnutrition Universal Screening Tool (MUST) protocol [24].

### Categorisation of Data

Pre-fracture mobility status was categorised as: (1) freely mobile without aids; (2) mobile outdoors with one aid; (3) mobile outdoors with two aids or frame, or limited to indoors (includes those with some mobility but never goes outside without help and no functional mobility) [21–23]. Early mobility, defined as the ability to sit or stand out of bed on the day of, or the day after, hip fracture surgery [25], was categorised into three groups: (1) ability to mobilise independently; (2) mobilised with assistance; and (3) unable to mobilise within 1-day of hip fracture surgery. Composite variables combining different levels of pre-fracture mobility and early mobility were also created: (1) freely mobile without aids and ability to mobilise independently; (2) mobile outdoors with one aid or mobilised with assistance; and (3) limited to indoors and unable to mobilise within 1-day of hip fracture surgery. The nutritional status based on MUST score of 0 indicates well-nourishment, 1 indicates “medium risk,” and ≥ 2 indicates “high risk” of malnutrition [24].

A change in discharge destination was defined as those who were admitted from their own home and survived to discharge but did not return home directly, including rehabilitation units, residential or nursing care. Prolonged LOS was defined as hospitalisation of ≥ 16 days (upper quartile). Types of hip fracture were grouped into intracapsular and extracapsular fractures. Elapsed (waiting) time to surgery ≥ 36 h from arrival in an emergency department was considered as a delay to surgery [21]. An ASA score ≥ 3 indicates severe systemic disease [26], and AMTS score ≤ 6 suggests moderate to severe cognitive impairment (possible delirium or dementia) [27].

### Statistical Analysis

Data for normally distributed data were presented as mean and standard deviation and skewed data as median and interquartile range. Pre-fracture mobility and early mobility (explanatory variables) were regressed simultaneously in a multivariable logistic regression model to predict outcomes (mortality, pressure ulcers and LOS), and discharge destinations (home, rehabilitation or residential/nursing care). Data are expressed as odds ratios (OR) and 95% confidence interval (CI). Two models were presented, model-1: unadjusted, and model-2: adjusted for age, sex, ASA, type of fracture, delay to surgery, and AMTS. Goodness-of-fit for a logistic regression model was assessed by Hosmer-Lemeshow tests. All analyses were conducted using IBM SPSS Statistics, v30.0 (IBM Corp., Armonk, NY).

## RESULTS

### General Characteristics

The mean (± SD) age of participants was 84.1 years (± 9.5) and median LOS was 10.1 days (interquartile range: 7–16), with 71.2% of patients were women. Most patients were from their own home (82.3%), followed by residential care (10.5%), nursing care (6.8%) and a small proportion from hospitals (0.4%). Prior to sustaining hip fractures, 38.0% mobilised freely without aids, 19.9% mobilised outdoors with one aid, and 42.1% mobilised outdoors with two aids/frame, or were limited to indoors, or had no functional mobility. In-hospital mortality was 5.8%, LOS ≥ 16 days 25.1% and pressure ulcers 2.2%. Amongst those who came from their own home, 54.5% were discharged back to their home, 31.5% to rehabilitation units, and 4.7% to residential or nursing care. The remaining patients either died (5.3%) or were discharged to other places or to unknown destinations (3.9%). The distributions of ASA, type of hip fracture, surgery delay, AMTS scores, and nutrition status are shown in Table 1.

**Table 1:** Characteristics of 3134 patients admitted with hip fractures from 1st April 2014–3rd June 2022 aged 60–107 years

	*n*	%
*Sex*		
Women	2230	71.2
Men	904	28.8
*Admitted from*		
Own home	2579	82.3
Residential care	329	10.5
Nursing care	212	6.8
Hospitals	14	0.4
*Pre-fracture mobility*		
Freely without aids	1191	38.0
Outdoors with one aid	623	19.9
Outdoors with two aids/frame or indoors	1320	42.1
*Early mobility*		
Independently	585	18.7
Assisted	1977	63.1
Immobile	572	18.3
*Outcomes in hospital*		
Death	181	5.8
Pressure ulcers	68	2.2
Length of stay ≥ 16 days (upper quartile)	788	25.1
*Discharge destinations for patients who came from own home*		
Rehabilitation unit	812	31.5
Residential care	59	2.3
Nursing care	63	2.4
Back to own home	1406	54.5
Death	137	5.3
Other or unknown	102	3.9
*ASA grades*		
1: Normal healthy individual	70	2.2
2: Mild systemic disease that does not limit activity	845	27.0
3: Severe systemic disease that limits activity but is not incapacitating	1797	57.3
4: Incapacitating systemic disease which is constantly life-threatening	383	12.2
5: Moribund - not expected to survive 24 h with or without surgery	7	0.2
Missing	32	1.0
*Type of fracture*		
Intracapsular	1787	57.0
Extracapsular hip fracture	1347	43.0
*Elapsed time to surgery*		
< 36 h	2562	81.7
≥ 36 h (delayed)	552	17.6
Missing	20	0.6
*Cognitive function*		
Abbreviated Mental Test	3098	98.9
Missing	36	1.1
*Nutritional status*		
Well-nourished	1467	46.8
Medium of malnutrition	743	23.7
High risk of malnutrition	216	6.9
Missing	708	22.6

ASA, American Society of Anesthesiologists; AMTS, Abbreviated Mental Test

**Fig. 1:**
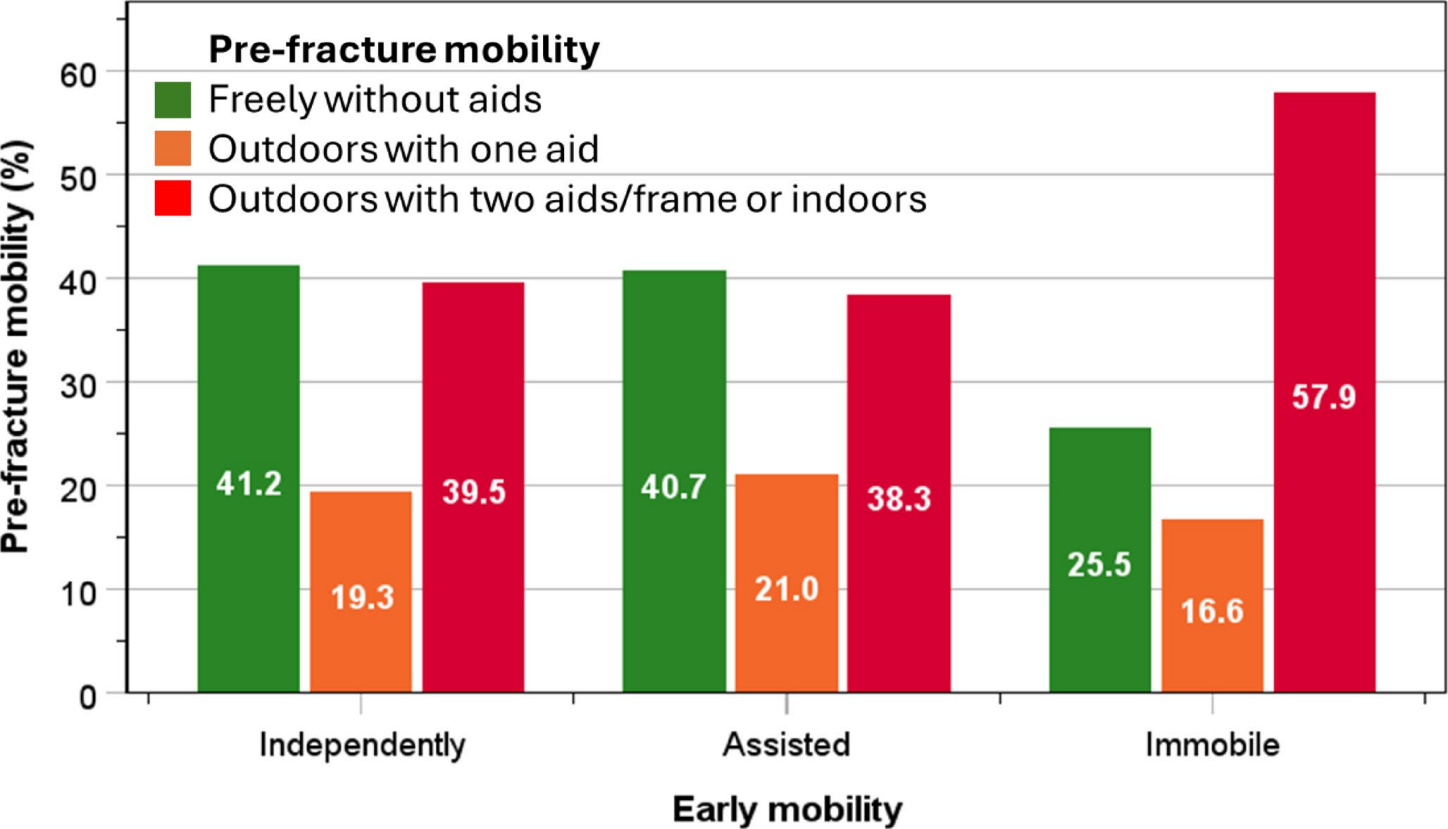
Proportions of patients with different levels of pre-fracture mobility and early post-surgical mobility

The proportions of post-surgical patients who were able to mobilise early independently (41.2%) or with assistance (40.7%) were higher amongst patients who were able to mobilise freely without aids prior to hip fractures than those in other pre-fracture categories. By contrast, the highest proportion of patients who were unable to mobilise early (57.9%) were amongst those who had limited mobility before hip fracture (Fig. 1).

**Fig. 2:**
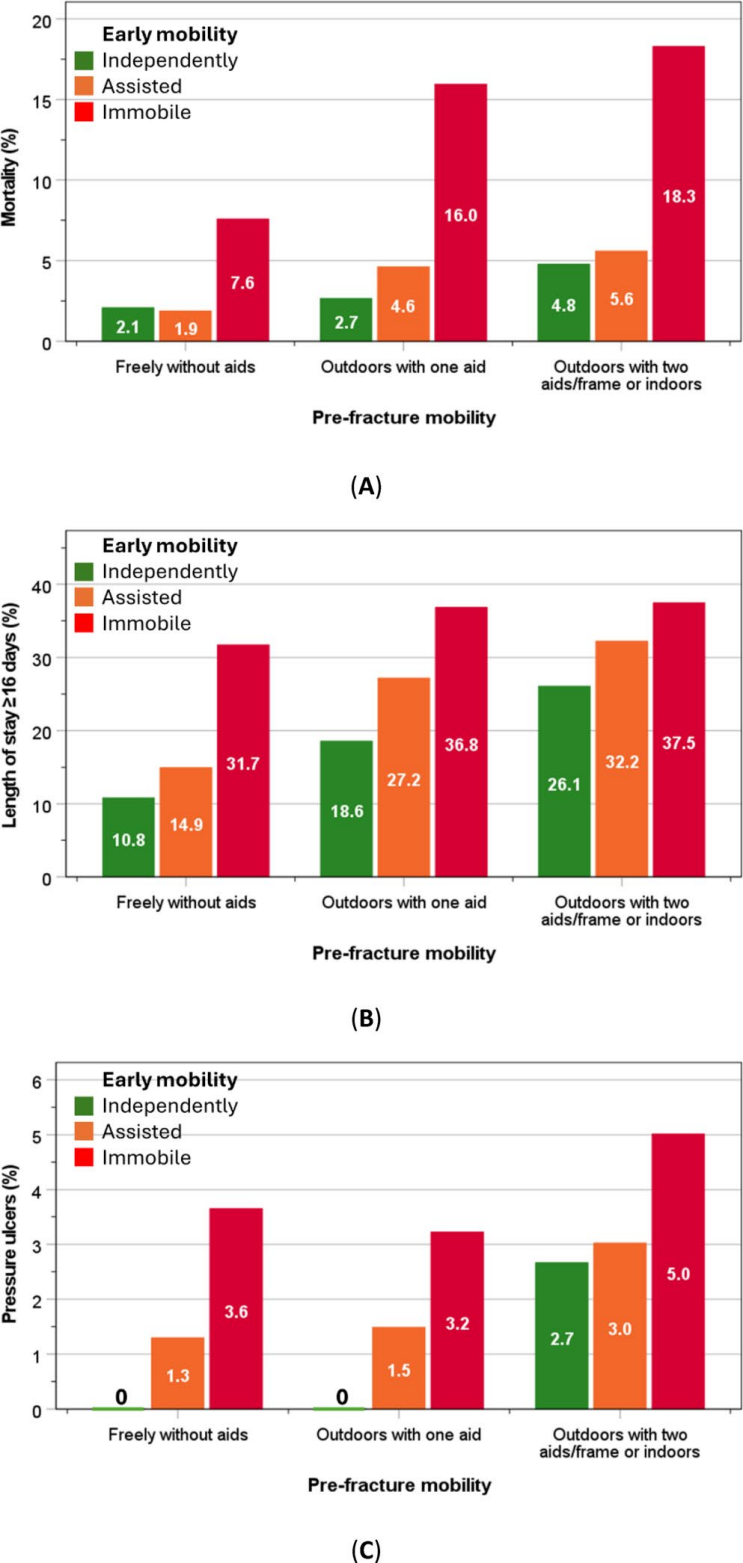
In-hospital outcomes including in-hospital mortality (**A**), LOS ≥16 days (upper quartile) (**B**) and pressure ulcers (**C**)

Within each pre-fracture mobility level, limited early mobility was associated with higher rates of mortality, LOS ≥ 16 days, and pressure ulcers, and these rates accentuated with poor pre-fracture mobility. Thus, the highest rates of poor outcomes were observed in those with pre-fracture mobility and limited early mobility (Fig. 2A–C).

**Fig. 3:**
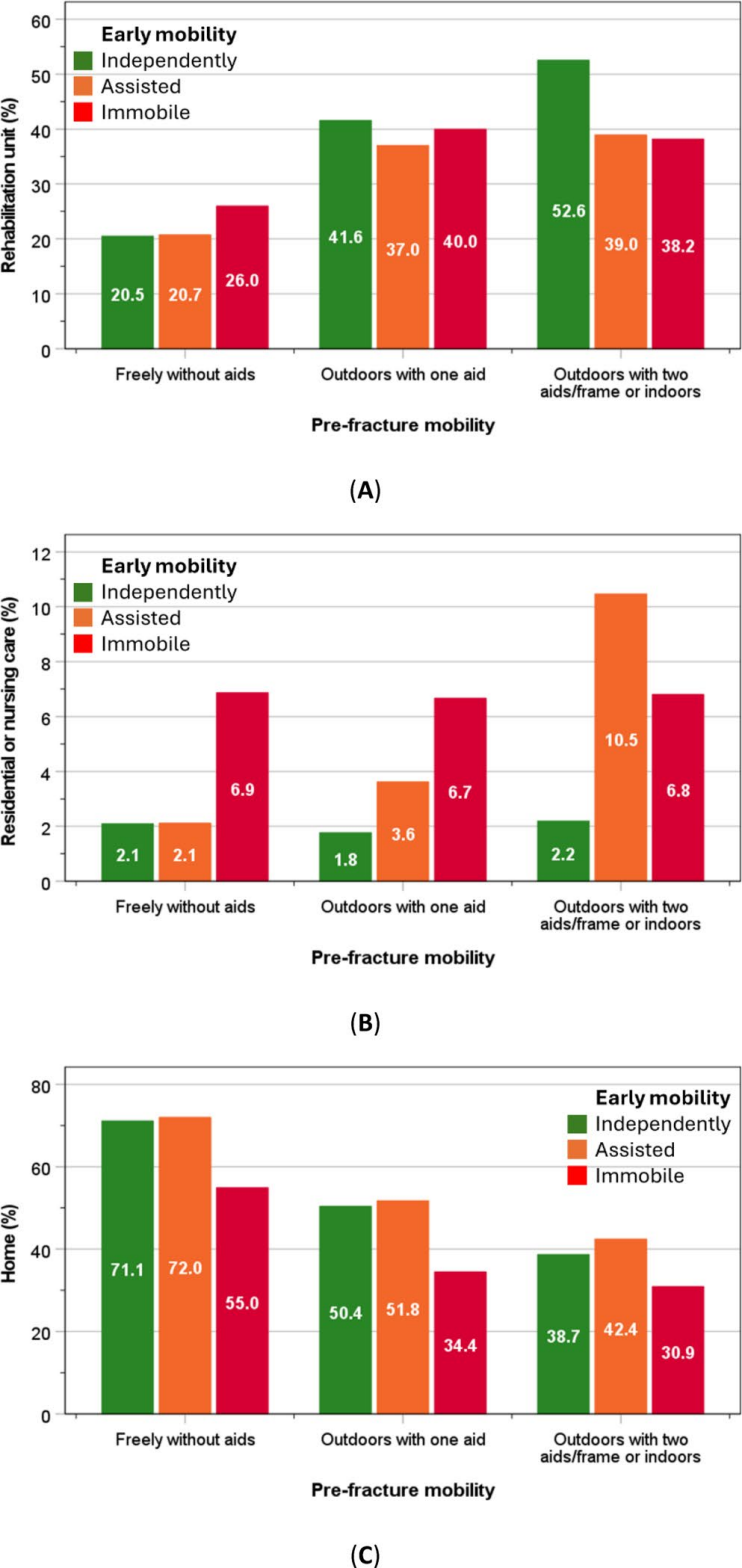
Discharge destinations to rehabilitation unit (**A**), residential/nursing care (**B**), or own home (**C**) amongst patients who were admitted from their own home

By contrast, rehabilitation was most suitable for those with the ability to mobilise early (within 1-day of hip fracture surgery). Whilst most of those who needed assistance for early mobility were placed in new residential/nursing care. On the other hand, the rates of discharge back to own homes were lower with limited early mobility and lowest in those with pre-existing poor pre-fracture mobility (Fig. 3A–C).

**Fig. 4:**
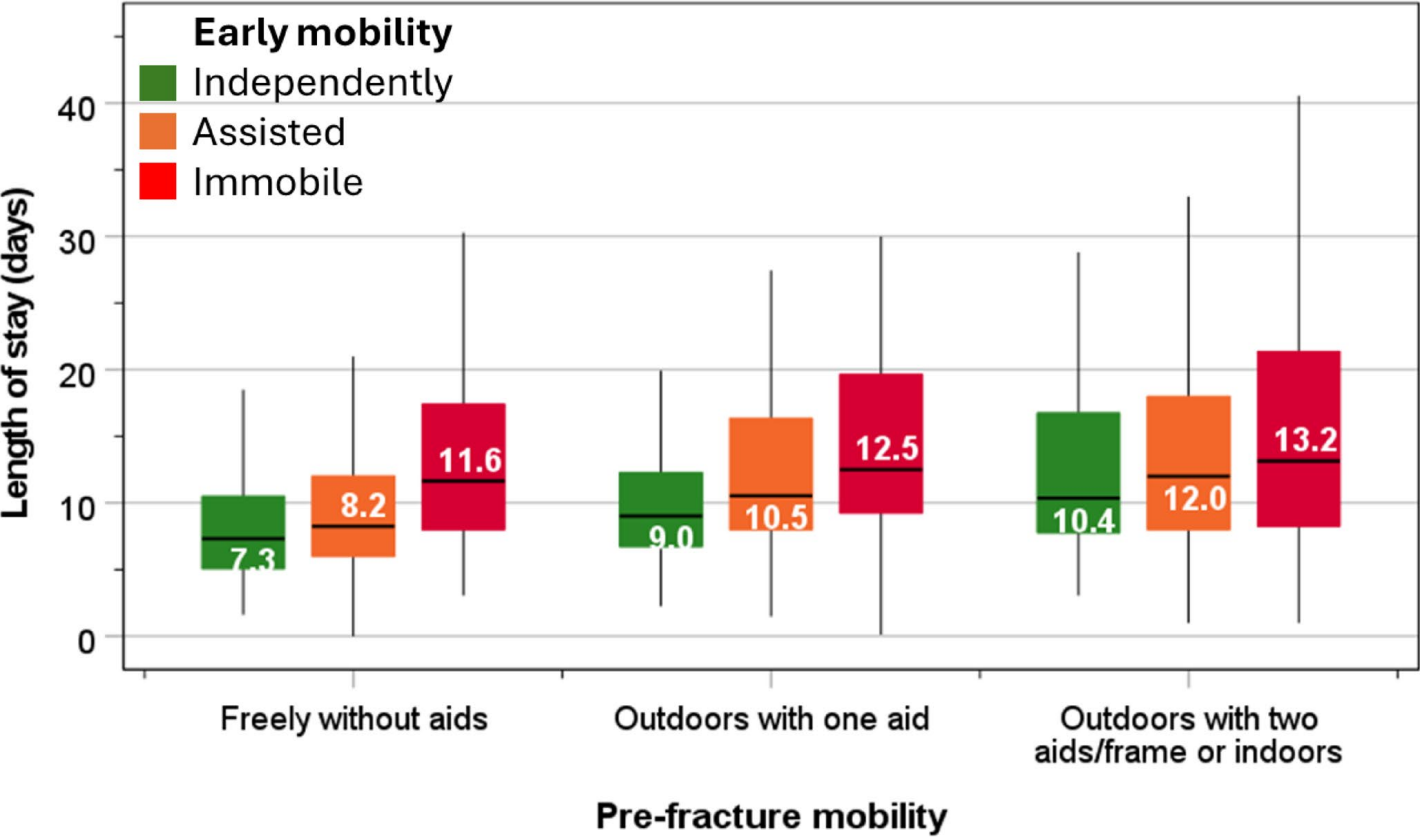
Box and whisker plot showing LOS according to pre-fracture mobility and early mobility after surgery. Median, interquartile range and 95% confidence intervals are denoted by horizontal bars, boxes, and whiskers respectively

The LOS in hospital increased progressively with poor pre-fracture mobility as well as limited early mobility within 1-day of hip fracture surgery. Within the group of patients who mobilised freely before fracture, the LOS for those who were able mobilised early independently after fracture was 7.3 days, rising to 8.2 days for those who mobilised with assistance, and 11.6 days for those who were unable to mobilise early. The corresponding values within the group who mobilised with one aid before hip fracture were 9.0 day, 10.5 days, and 12.5 days, and within the group who mobilised with two aids or limited to indoors were increased further to 10.4 days, 12.0 days and 13.2 days (Fig. 4).

The degree of severity of pre-fracture mobility and early mobility within one day of hip surgery, as well as their combination (composite variable) was associated with poorer health indicators including ASA, type of hip fracture, AMTS scores, and nutrition status (Table 2).

**Table 2:** The associations of pre-fracture mobility and early mobility with health indicators

	Health indicators							
	ASA grade ≥ 3		AMTS score ≤ 6		Extracapsular fracture		Risk of malnutrition*	
	%	χ^2^ (*P*)	%	χ^2^ (*P*)	%	χ^2^ (*P*)	%	χ^2^ (*P*)
*Pre-fracture mobility*								
Freely without aids (reference)	51.7	356 (**< 0.001**)	17.4	216 **(< 0.001**)	38.5	16.2 (**< 0.001)**	29.4	79.6 (**< 0.001**)
Outdoors with one aid	72.2		20.1		45.1		37.8	
Outdoors with two aids/frame or indoors	86.1		42.4		46.1		49.1	
*Early mobility*								
Independently (reference)	67.7	39.1 (**< 0.001**)	24.0	32.4 (**< 0.001**)	40.2	2.3 (**0.314**)	26.7	54.5 (**< 0.001**)
Assisted	67.9		27.0		43.7		44.4	
Immobile	80.9		37.9		43.5		40.9	
*Composite of pre-fracture and early mobility*								
Freely without aids and Independently	45.2	134 (**< 0.001**)	7.1	113 (**< 0.001**)	33.6	10.1 (**0.007**)	16.6	63.6 (**< 0.001**)
Outdoors with one aid or Assisted	70.1		28.0		43.5		41.2	
Outdoors with two aids/frame or indoors and Immobility	89.7		47.6		45.9		48.4	

Bold type indicates a statistical significant relationship

*n = 2426 (no information available for 708 cases); ASA, American Society of Anesthesiologists; AMTS, Abbreviated Mental Test

Tables 3 and 4 show findings from multivariable logistic regression where all predictor variables were regressed simultaneously to adjust for each other including pre-fracture mobility, early mobility, age and sex. Compared to patients who mobilised freely before hip fracture, those who mobilised with one aid before fracture had increased risk of death and longer stay, less likely to return to own home, more likely to be transferred to a rehabilitation unit in hospital independent of early mobility, age and sex. Compared to patients who were able to mobilise early, those who needed assistance to mobilise early were likely to stay longer in hospital, be transferred to a rehabilitation unit and residential/nursing care, independent of pre-fracture mobility, age and sex, ASA, type of fracture, delay to surgery, and AMTS (Table 3).

**Table 3:** Multivariable logistic regression assessing pre-fracture mobility and early mobility within one day of hip surgery to predict healthcare outcomes

	Death			LOS ≥ 16 days			Pressure ulcers		
	**OR**	**95% CI**	***P***	**OR**	**95% CI**	***P***	**OR**	**95% CI**	***P***
**Model 1: Unadjusted**									
*Pre-fracture mobility*									
Freely without aids (reference)	1	–	–	1	–	–	1	–	–
Outdoors with one aid	2.26	1.38–3.70	**0.001**	1.90	1.50–2.40	**< 0.001**	1.09	0.47–2.51	0.842
Outdoors with two aids/frame or indoors	2.83	1.88–4.28	**< 0.001**	2.34	1.93–2.85	**< 0.001**	2.39	1.31–4.35	**0.004**
*Early mobility*									
Independently (reference)	1	–	–	1	–	–	1	–	–
Assisted	1.20	0.72–2.00	0.488	1.43	1.13–1.81	**0.003**	1.94	0.81–4.61	0.135
Immobile	4.66	2.78–7.80	**< 0.001**	2.23	1.69–2.93	**< 0.001**	3.70	1.49–9.17	**0.005**
*Composite of pre-fracture and early mobility*									
Freely without aids and Independently (reference)	1	–	–	1	–	–	–*	–	–
Outdoors with one aid or Assisted	2.26	1.38–3.70	**0.001**	1.20	0.72-2.00	0.488	–	–	–
Outdoors with two aids/frame or indoors and Immobility	2.83	1.88–4.28	**< 0.001**	4.66	2.78–7.80	**< 0.001**	–	–	–
**Model 2: Adjusted for age**,** sex**,** ASA**,** fracture type**,** delay to surgery**,** and AMTS**									
*Pre-fracture mobility*									
Freely without aids (reference)	1	–	–	1	–	–	1	–	–
Outdoors with one aid	1.54	0.92–2.57	0.101	1.51	1.18–1.94	**0.001**	1.02	0.43–2.40	0.965
Outdoors with two aids/frame or indoors	1.87	1.20–2.91	**0.006**	1.65	1.33–2.05	**< 0.001**	1.91	1.00-3.68	**0.050**
*Early mobility*									
Independently (reference)	1	–	–	1	–	–	1	–	–
Assisted	1.11	0.66–1.87	0.684	1.40	1.10–1.78	**0.006**	1.94	0.81–4.64	0.134
Immobile	4.17	2.47–7.05	**< 0.001**	2.05	1.55–2.72	**< 0.001**	3.31	1.33–8.26	**0.010**
*Composite of pre-fracture and early mobility*									
Freely without aids and Independently (reference)	1	–	–	1	–	–	–*	–	–
Outdoors with one aid or Assisted	1.52	0.60–3.83	0.374	1.94	1.27–2.98	**0.002**	–	–	–
Outdoors with two aids/frame or indoors and Immobility	5.84	2.24–15.24	**< 0.001**	2.78	1.71–4.51	**< 0.001**	–	–	–

Bold type indicates a statistical significant relationship

ASA, American Society of Anesthesiologists; AMTS, Abbreviated Mental Test

*Too few pressure ulcers for analysis with composite variables

**Table 4:** Multivariable logistic regression including pre-fracture mobility, early mobility after surgery, age and sex to predict discharge destinations

	Rehabilitation			Residential/nursing care			Home		
	**OR**	**95% CI**	***P***	**OR**	**95% CI**	***P***	**OR**	**95% CI**	***P***
**Model 1: Unadjusted**									
*Pre-fracture mobility*									
Freely without aids (reference)	1	–	–	1	–﻿	–	1	–	–
Outdoors with one aid	2.31	1.85–2.87	**< 0.001**	1.39	0.79-2.44	0.247	0.42	0.34–0.51	**< 0.001**
Outdoors with two aids/frame or indoors	2.58	2.11–3.15	**< 0.001**	3.15	2.03–4.90	**< 0.001**	0.29	0.24–0.35	**< 0.001**
*Early mobility*									
Independently (reference)	1	–﻿	–﻿	1	–	–	1	–	–
Assisted	0.79	0.64–0.99	**0.040**	2.48	1.27–4.82	**0.008**	1.08	0.88-1.34	0.468
Immobile	0.86	0.65–10.15	0.311	2.87	1.37–6.01	**0.005**	0.57	0.43–0.76	**< 0.001**
*Composite of pre-fracture and early mobility*									
Freely without aids and Independently (reference)	1	–	–	1	–	–	1	–	–
Outdoors with one aid or Assisted	1.86	1.34–2.58	**< 0.001**	2.40	0.97–5.95	0.058	0.50	0.37–0.67	**< 0.001**
Outdoors with two aids/frame or indoors and Immobility	2.40	1.56–3.68	**< 0.001**	3.42	1.20–9.76	**0.022**	0.18	0.12–0.27	**< 0.001**
**Model 2: Adjusted for age**,** sex**,** ASA**,** fracture type**,** delay to surgery**,** and AMTS**									
*Pre-fracture mobility*									
Freely without aids (reference)	1	–	–	1	–	–	1	–	–
Outdoors with one aid	1.89	1.50–2.38	**< 0.001**	1.15	0.64–2.05	0.637	0.56	0.45–0.70	**< 0.001**
Outdoors with two aids/frame or indoors	1.99	1.60–2.48	**< 0.001**	2.26	1.40–3.64	**< 0.001**	0.43	0.35–0.53	**< 0.001**
*Early mobility*									
Independently (reference)	1	–	–	1	–	–	1	–	–
Assisted	0.78	0.63–0.98	**0.033**	2.46	1.26–4.80	**0.008**	1.11	0.89–1.38	0.361
Immobile	0.86	0.64–1.14	0.291	2.95	1.40–6.22	**0.004**	0.58	0.43–0.77	**< 0.001**
*Composite of pre-fracture and early mobility*									
Freely without aids and Independently (reference)	1	–	–	1	–	–	1	–	–
Outdoors with one aid or Assisted	1.43	1.02–2.00	**0.039**	1.67	0.67–4.21	0.274	0.71	0.52–0.97	**0.030**
Outdoors with two aids/frame or indoors and Immobility	1.60	1.02–2.50	**0.040**	2.08	0.71–6.10	0.181	0.31	0.20–0.47	**< 0.001**

Bold type indicates a statistical significant relationship

ASA, American Society of Anesthesiologists; AMTS, Abbreviated Mental Test

Compared to patients who mobilised freely before fractures, those who mobilised with two aids or were limited to indoors before a fracture were more likely to die, OR = 1.87 (1.20–2.91); stay in hospital ≥ 16days, OR = 1.65 (1.33–2.05); acquire pressure ulcers: OR = 1.91 (1.00-3.68) (Table 3); require rehabilitation: OR = 1.99 (1.60–2.48) and residential or nursing care; OR = 2.26 (1.40–3.64); and less likely to return home, OR = 0.43 (0.35–0.53) (Table 4). Similarly, compared to patients who mobilised early, those who were unable to mobilise early were more likely to die, OR = 4.17 (2.47–7.05), stay in hospital ≥ 16days, OR = 2.05 (1.55–2.72); develop pressure ulcers, OR = 3.31 (1.33–8.26) (Table 3); and require residential or nursing care, OR = 2.95 (1.40–6.22); and less likely to return home, OR = 0.58 (0.43–0.77) (Table 4). Hosmer-Lemeshow tests showed logistic regression models in this study were a good fit for the observed data (*P* > 0.05).

Composite variables created from the combination of pre-fracture and early mobility showed that compared to those who mobilised freely without aids prior to fracture and mobilised early independently, those who mobilised outdoors with two aids/frame or indoors prior to fracture and were unable to mobilise early had an accentuated risk of poorer outcomes than individual mobility measures, including mortality: OR = 5.84 (2.24–15.24), staying in hospital ≥ 16 days: OR = 2.78 (1.71–4.51), or not returning to own home: OR = 0.31 (0.20–0.47) (Tables 3 and 4). Analysis of men and women separately showed similar trends for the associations of pre-fracture and early mobility with outcomes. The ORs were generally higher in men than women (Supplementary Tables 1 & 2).

## Discussion

### Key Findings

The present study shows that early mobility within 1-day of hip fracture surgery was in part dependant on pre-fracture mobility. In turn, pre-fracture mobility and early mobility have influences on outcome measures, independent from each other as well as from age and sex. Furthermore, the risk of poor outcome measures is accentuated amongst patients with limited early mobility as well as pre-fracture mobility. These key performance indicators are useful for identifying patients with potential for remobilisation and could also be served as prognosticators of poor outcomes, and are thus helpful for multidisciplinary team review of care management and discharge planning for patients admitted with hip fractures. Evidence from our study that even among people with pre-fracture mobility limitations, early mobilization improves outcomes, suggests that there should be a more concentrated effort to early mobilise these patients. For example, being more intentional with individualised goal setting; or employing interventions to mitigate post operative complications such as delirium to improve functional outcomes and recovery.

Findings of a close relationship between pre-fracture mobility and early mobility observed in this study are consistent with data from previous reports [28–30]. Early mobilisation after hip surgery, recommended by the National Institute for Health and Care Excellence (NICE) for improving healthcare outcomes [31], has variably been defined by different countries and researchers [32]. However, it is nationally standardised by the NHFD in the UK as mobilisation within 1-day of hip fracture surgery [25, 33], which is used in this study. Findings from this study of the association of early mobility with a range of outcomes including mortality and prolonged LOS are broadly in-line with previous observations: these include earlier discharge from hospital (shorter LOS in hospital) [15, 16] and faster functional recovery (walking) [17–19]. In this study, we have also demonstrated that pre-fracture mobility also has an independent and important role in outcome measures, and together with early mobility, provides a better discrimination of outcomes. Overall, patients who were able to mobilise early and were also able to mobilise freely before their fracture had the lowest risk of poor outcomes. By contrast, those who had limited ability to mobilise both early after surgery and before fracture had the worst outcomes. Furthermore, we have also shown that limitation in both pre-fracture mobility and early mobility had negative impacts on pressure ulcers. This complication can lead to delays in discharge from hospital [34].

The observation that patients who were able to mobilise early were more likely to return to their own home is also consistent with previous findings [17, 19]. However, unique to this study, a pre-fracture ability to mobilise freely was also a significant indicator of returning home. Both pre-fracture and early mobility were also independent indicators of suitability for rehabilitation. Those with more limited pre-fracture mobility were still being considered for rehabilitation: however, those with limited early mobility by contrast were less likely to be considered for rehabilitation. On the other hand, limitations in both pre-fracture mobility and early mobility are significant indicators of the need for higher level of care – requiring new placement for residential/nursing care.

This study also found that intermediate pre-fracture mobility (i.e. with one aid) or early mobility with assistance groups are important. These groups comprise a large proportion of individuals who also require higher level of care such as rehabilitation or residential/nursing care and therefore incur substantial costs to healthcare services. In clinical practice, patients are routinely assessed by physiotherapists to set achievable goals, determined by pre-fracture mobility levels. Patients with limited mobility before fractures have less potential for early mobilisation. Whilst those with the ability to mobilise independently prior to hip fractures should be encouraged to mobilise as early as possible so a decision for rehabilitation could be made more rapidly to improve recovery and avoid discharge delays.

### Validity of Mobility Measures

There are several validated scales for assessing physical functioning including New Mobility Score [35] and Clinical Frailty Scale [36]. The mobility measure used in our study is a key indicator of physical functioning adopted by the NHFD audit protocol [22, 23]. To enhance its validity, we extended the analysis to relate to key health indicators including ASA, AMTS, type of fracture and nutritional status. Poor pre-fracture and early mobility, and their combination as composite variables, were consistently associated with worse health indicators. However, it would be of interest to directly compare the performance of different scales in future studies.

### Limitations

Due to its cross-sectional design in a single centre, findings from our study therefore should be interpreted with caution. However, the data are comparable to those of national data in England and Wales [22, 23]. The few missing cases (< 4%) from certain variables (mainly confounding factors) were likely to have little influences on findings from our study. However, there were 22.6% of missing cases for nutritional status therefore this factor was not considered as potential confounding factor in multivariable logistic regression. Since men are at higher risk of worse outcomes, we also performed the stratification technique to examine men and women separately, but this approach diminished the sample size, particularly in men, therefore findings were not reliable for certain outcomes such as pressure ulcers or discharge destination to residential or nursing care. Larger studies are therefore necessary to examine sex-specific associations between mobility and outcomes. Patients with a recurrent fracture may have worse outcomes and therefore some bearing on our findings. However, we did not have this information.

The strengths of this study include reliable data procurement which were collected consecutively according to NHFD protocols, including the use of standardised scales for pre-fracture and early mobility, and a relatively large number of patients derived from a single NHS hospital that serves a population of > 400,000 people.

In conclusion, pre-fracture mobility and early mobility within 1-day of hip fracture surgery are independent indicators of health outcomes and level of care; thus, both indicators should be taken into consideration by multidisciplinary team upon reviewing management and discharge planning for adults undergoing hip fracture surgery.

## Supplementary Information

Below is the link to the electronic supplementary material.

Supplementary Material 1.

## Declarations

### Human and Animal Participants

This article does not contain any studies with animals performed by any of the authors. Informed consent Informed consent was obtained from all individual participants included in the study.
