# Supplementary material for: Influences of Pre-fracture Mobility and Early Mobility on Healthcare Outcome Measures in Older Patients Undergoing Hip Fracture Surgery
Source: Calcif Tissue Int. 2026 Jan 23;117(1):12. doi: 10.1007/s00223-025-01475-6 (PMC12827345; doi:10.1007/s00223-025-01475-6)
Supplement: Supplementary file 1 — Supplementary Material 1. [file 223_2025_1475_MOESM1_ESM.docx]

**Supplementary table 1.** Sex-specific multivariable logistic regression assessing pre-fracture mobility and early mobility within one day of hip surgery to predict healthcare outcomes, unadjusted(**A**) and adjusted (**B**).

|  | **(A) Multivariable logistic regression (unadjusted)** | | | | | | | | |
| --- | --- | --- | --- | --- | --- | --- | --- | --- | --- |
| **MEN** | **Death** | | | **LOS ≥16 days** | | | **Pressure ulcers** | | |
| *Pre-fracture mobility* | **OR** | **95% CI** | ***P*** | **OR** | **95% CI** | ***P*** | **OR** | **95% CI** | ***P*** |
| Freely without aids (reference) | 1 | **--** | **--** | 1 | **--** | **--** | 1 | **--** | **--** |
| Outdoors with one aid | 2.26 | 1.14-4.48 | **0.020** | 1.84 | 1.23-2.74 | **0.003** | 0.76 | 0.22-2.58 | 0.659 |
| Outdoors with two aids/frame or indoors | 2.61 | 1.43-4.78 | **0.002** | 2.39 | 1.69-3.38 | **<0.001** | 1.09 | 0.42-2.82 | 0.854 |
| *Early mobility* |  |  |  |  |  |  |  |  |  |
| Independently (reference) | 1 | **--** | **--** | 1 | **--** | **--** | 1 | **--** | **--** |
| Assisted | 1.16 | 0.52-2.57 | 0.717 | 1.55 | .99-2.42 | 0.053 | 3.08 | 0.39-24.05 | 0.284 |
| Immobile | 4.33 | 1.95-9.64 | **<0.001** | 2.46 | 1.49-4.05 | **<0.001** | 9.04 | 1.14-71.61 | **0.037** |
| *Composite of pre-fracture and early mobility* |  |  |  |  |  |  |  |  |  |
| Freely without aids **and** Independently (reference) | 1 | **--** | **--** | 1 | **--** | **--** | **--*** | **--** | **--** |
| Outdoors with one aid **or** Assisted | 1.76 | 0.53-5.77 | 0.354 | 4.51 | 1.93-10.59 | **<0.001** | **--** | **--** | **--** |
| Outdoors with two aids/frame or indoors **and** Immobility | 8.11 | 2.36-27.88 | **<0.001** | 6.73 | 2.67-16.96 | **<0.001** | **--** | **--** | **--** |
| **WOMEN** |  |  |  |  |  |  |  |  |  |
| *Pre-fracture mobility* |  |  |  |  |  |  |  |  |  |
| Freely without aids (reference) | 1 | **--** | **--** | 1 | **--** | **--** | 1 | **--** | **--** |
| Outdoors with one aid | 2.12 | 1.03-4.35 | **0.041** | 1.90 | 1.42-2.55 | **<0.001** | 1.39 | 0.44-4.42 | 0.574 |
| Outdoors with two aids/frame or indoors | 3.29 | 1.85-5.85 | **<0.001** | 2.38 | 1.88-3.02 | **<0.001** | 3.90 | 1.70-8.93 | **0.001** |
| *Early mobility* |  |  |  |  |  |  |  |  |  |
| Independently (reference) | 1 | **--** | **--** | 1 | **--** | **--** |  |  |  |
| Assisted | 1.19 | 0.61-2.34 | 0.612 | 1.37 | 1.03-1.81 | **0.028** | 1.74 | 0.67-4.56 | 0.259 |
| Immobile | 4.41 | 2.23-8.70 | **<0.001** | 2.05 | 1.47-2.86 | **<0.001** | 2.53 | 0.89-7.20 | 0.083 |
| *Composite of pre-fracture and early mobility* |  |  |  |  |  |  |  |  |  |
| Freely without aids **and** Independently (reference) | 1 | **--** | **--** | 1 | **--** | **--** | **--*** | **--** | **--** |
| Outdoors with one aid **or** Assisted | 3.02 | 0.73-12.45 | 0.126 | 2.25 | 1.39-3.63 | **<0.001** | **--** | **--** | **--** |
| Outdoors with two aids/frame or indoors **and** Immobility | 14.08 | 3.33-59.65 | **<0.001** | 4.38 | 2.56-7.51 | **<0.001** | **--** | **--** | **--** |

ASA, American Society of Anesthesiologists; AMTS, Abbreviated Mental Test.*Too few pressure ulcers for analysis with composite variables.

|  | **(B) Multivariable logistic regression (adjusted for age, ASA, fracture type, delay to surgery, and AMTS)** | | | | | | | | |
| --- | --- | --- | --- | --- | --- | --- | --- | --- | --- |
| **MEN** | **Death** | | | **LOS ≥16 days** | | | **Pressure ulcers** | | |
| *Pre-fracture mobility* | **OR** | **95% CI** | ***P*** | **OR** | **95% CI** | ***P*** | **OR** | **95% CI** | ***P*** |
| Freely without aids (reference) | 1 | **--** | **--** | 1 | **--** | **--** | 1 | **--** | **--** |
| Outdoors with one aid | 1.70 | .83-3.46 | .147 | 1.42 | .93-2.18 | .103 | .56 | .16-1.97 | .366 |
| Outdoors with two aids/frame or indoors | 1.79 | .94-3.42 | .076 | 1.78 | 1.21-2.60 | .003 | .65 | .24-1.79 | .408 |
| *Early mobility* |  |  |  |  |  |  |  |  |  |
| Independently (reference) | 1 | **--** | **--** | 1 | **--** | **--** | 1 | **--** | **--** |
| Assisted | 1.11 | .49-2.48 | .808 | 1.48 | .94-2.32 | .091 | 2.74 | .35-21.66 | .338 |
| Immobile | 4.38 | 1.94-9.85 | <.001 | 2.29 | 1.38-3.82 | .001 | 7.55 | .95-60.11 | .056 |
| *Composite of pre-fracture and early mobility* |  |  |  |  |  |  |  |  |  |
| Freely without aids **and** Independently (reference) | 1 | **--** | **--** | 1 | **--** | **--** | **--*** | **--** | **--** |
| Outdoors with one aid **or** Assisted | 1.24 | .37-4.20 | .728 | 3.40 | 1.43-8.10 | .006 | **--** | **--** | **--** |
| Outdoors with two aids/frame or indoors **and** Immobility | 5.24 | 1.46-18.84 | .011 | 4.34 | 1.67-11.24 | .003 | **--** | **--** | **--** |
| **WOMEN** |  |  |  |  |  |  |  |  |  |
| *Pre-fracture mobility* |  |  |  |  |  |  |  |  |  |
| Freely without aids (reference) | 1 | **--** | **--** | 1 | **--** | **--** | 1 | **--** | **--** |
| Outdoors with one aid | 1.39 | .66-2.96 | .388 | 1.56 | 1.15-2.13 | .005 | 1.55 | .48-5.06 | .465 |
| Outdoors with two aids/frame or indoors | 1.92 | 1.03-3.56 | .040 | 1.60 | 1.23-2.08 | <.001 | 3.85 | 1.55-9.52 | .004 |
| *Early mobility* |  |  |  |  |  |  |  |  |  |
| Independently (reference) | 1 | **--** | **--** | 1 | **--** | **--** |  |  |  |
| Assisted | 1.15 | .58-2.27 | .688 | 1.37 | 1.03-1.82 | .030 | 1.82 | .69-4.78 | .225 |
| Immobile | 4.16 | 2.09-8.29 | <.001 | 1.94 | 1.38-2.72 | <.001 | 2.34 | .81-6.77 | .116 |
| *Composite of pre-fracture and early mobility* |  |  |  |  |  |  |  |  |  |
| Freely without aids **and** Independently (reference) | 1 | **--** | **--** | 1 | **--** | **--** | **--*** | **--** | **--** |
| Outdoors with one aid **or** Assisted | 1.92 | .46-8.05 | .375 | 1.54 | .94-2.52 | .087 | **--** | **--** | **--** |
| Outdoors with two aids/frame or indoors **and** Immobility | 6.90 | 1.57-30.25 | .010 | 2.33 | 1.32-4.09 | .003 | **--** | **--** | **--** |

ASA, American Society of Anesthesiologists; AMTS, Abbreviated Mental Test.*Too few pressure ulcers for analysis with composite variables.

**Supplementary table 2.** Sex-specific multivariable logistic regression assessing pre-fracture mobility and early mobility within one day of hip surgery to predict discharge destinations, unadjusted(**A**) and adjusted (**B**).

|  | 1. **Multivariable logistic regression (unadjusted)** | | | | | | | | |
| --- | --- | --- | --- | --- | --- | --- | --- | --- | --- |
| **MEN** | **Rehabilitation** | | | **Residential/nursing care** | | | **Home** | | |
| *Pre-fracture mobility* | **OR** | **95% CI** | ***P*** | **OR** | **95% CI** | ***P*** | **OR** | **95% CI** | ***P*** |
| Freely without aids (reference) | 1 | **--** | **--** | 1 | **--** | **--** | 1 | **--** | **--** |
| Outdoors with one aid | 2.14 | 1.45-3.16 | **<0.001** | 0.70 | 0.21-2.30 | 0.553 | 0.55 | 0.39-0.80 | **0.001** |
| Outdoors with two aids/frame or indoors | 2.13 | 1.46-3.13 | **<0.001** | 2.07 | 0.87-4.92 | 0.100 | 0.39 | 0.27-0.56 | **<0.001** |
| *Early mobility* |  |  |  |  |  |  |  |  |  |
| Independently (reference) | 1 | **--** | **--** | 1 | **--** | **--** | 1 | **--** | **--** |
| Assisted | 0.64 | 0.42-0.96 | **0.030** | 4.36 | 0.57-33.34 | 0.157 | 1.36 | 0.92-2.01 | 0.127 |
| Immobile | 0.53 | 0.32-0.88 | **0.015** | 10.03 | 1.27-79.07 | **0.029** | 0.60 | 0.37-0.98 | **0.043** |
| *Composite of pre-fracture and early mobility* |  |  |  |  |  |  |  |  |  |
| Freely without aids **and** Independently (reference) | 1 | **--** | **--** | 1 | **--** | **--** | **--** | **--** | **--** |
| Outdoors with one aid **or** Assisted | 1.51 | 0.84-2.72 | 0.166 | --* | -- | **--** | 0.57 | 0.34-0.97 | **0.040** |
| Outdoors with two aids/frame or indoors **and** Immobility | 1.10 | 0.50-2.42 | 0.803 | --* | -- | **--** | 0.23 | 0.11-0.48 | **<0.001** |
| **WOMEN** |  |  |  |  |  |  |  |  |  |
| *Pre-fracture mobility* |  |  |  |  |  |  |  |  |  |
| Freely without aids (reference) | 1 | **--** | **--** | 1 | **--** | **--** | 1 | **--** | **--** |
| Outdoors with one aid | 2.40 | 1.84-3.13 | **<0.001** | 1.75 | 0.92-3.33 | 0.088 | 0.37 | 0.29-0.47 | **<0.001** |
| Outdoors with two aids/frame or indoors | 2.78 | 2.20-3.51 | **<0.001** | 3.54 | 2.11-5.95 | **<0.001** | 0.26 | 0.21-0.33 | **<0.001** |
| *Early mobility* |  |  |  |  |  |  |  |  |  |
| Independently (reference) | 1 | **--** | **--** | 1 | **--** | **--** |  |  |  |
| Assisted | .87 | 0.67-1.13 | 0.289 | 2.28 | 1.12-4.63 | **0.023** | 0.99 | 0.77-1.27 | 0.913 |
| Immobile | 1.10 | 0.78-1.56 | 0.570 | 2.03 | 0.88-4.70 | 0.098 | 0.57 | 0.40-0.80 | **0.001** |
| *Composite of pre-fracture and early mobility* |  |  |  |  |  |  |  |  |  |
| Freely without aids **and** Independently (reference) | 1 | **--** | **--** | 1 | **--** | **--** | **--** | **--** | **--** |
| Outdoors with one aid **or** Assisted | 2.03 | 1.37-3.01 | **<0.001** | 1.90 | 0.76-4.76 | 0.169 | 0.47 | 0.33-0.67 | **<0.001** |
| Outdoors with two aids/frame or indoors **and** Immobility | 3.44 | 2.05-5.79 | **<0.001** | 1.99 | 0.62-6.41 | 0.251 | 0.16 | 0.10-0.27 | **<0.001** |

ASA, American Society of Anesthesiologists; AMTS, Abbreviated Mental Test.*Too few pressure ulcers for analysis with composite variables.

|  | **(B) Multivariable logistic regression (adjusted for age, ASA, fracture type, delay to surgery, and AMTS)** | | | | | | | | |
| --- | --- | --- | --- | --- | --- | --- | --- | --- | --- |
| **MEN** | **Rehabilitation** | | | **Residential/nursing care** | | | **Home** | | |
| *Pre-fracture mobility* | **OR** | **95% CI** | ***P*** | **OR** | **95% CI** | ***P*** | **OR** | **95% CI** | ***P*** |
| Freely without aids (reference) | 1 | **--** | **--** | 1 | **--** | **--** | 1 | **--** | **--** |
| Outdoors with one aid | 1.56 | 1.03-2.37 | 0.037 | .72 | 0.20-2.52 | 0.605 | 0.84 | 0.57-1.25 | 0.387 |
| Outdoors with two aids/frame or indoors | 1.56 | 1.03-2.36 | 0.036 | 1.85 | 0.72-4.80 | 0.204 | 0.62 | 0.42-0.92 | 0.018 |
| *Early mobility* |  |  |  |  |  |  |  |  |  |
| Independently (reference) | 1 | **--** | **--** | 1 | **--** | **--** | 1 | **--** | **--** |
| Assisted | 0.63 | 0.41-0.95 | 0.027 | 3.94 | 0.51-30.36 | 0.189 | 1.39 | 0.93-2.09 | 0.111 |
| Immobile | 0.49 | 0.29-0.83 | 0.008 | 9.46 | 1.19-74.90 | 0.033 | 0.61 | 0.36-1.01 | 0.053 |
| *Composite of pre-fracture and early mobility* |  |  |  |  |  |  |  |  |  |
| Freely without aids **and** Independently (reference) | 1 | **--** | **--** | 1 | **--** | **--** | **--** | **--** | **--** |
| Outdoors with one aid **or** Assisted | 1.15 | 0.62-2.11 | 0.658 | --* | -- | **--** | 0.81 | 0.46-1.41 | 0.453 |
| Outdoors with two aids/frame or indoors **and** Immobility | 0.68 | 0.30-1.54 | 0.352 | --* | -- | **--** | 0.40 | 0.19-0.84 | 0.017 |
| **WOMEN** |  |  |  |  |  |  |  |  |  |
| *Pre-fracture mobility* |  |  |  |  |  |  |  |  |  |
| Freely without aids (reference) | 1 | **--** | **--** | 1 | **--** | **--** | 1 | **--** | **--** |
| Outdoors with one aid | 2.03 | 1.53-2.69 | <0.001 | 1.36 | 0.70-2.64 | 0.358 | 0.47 | 0.36-0.62 | <0.001 |
| Outdoors with two aids/frame or indoors | 2.21 | 1.71-2.86 | <0.001 | 2.39 | 1.37-4.18 | 0.002 | 0.37 | 0.29-0.47 | <0.001 |
| *Early mobility* |  |  |  |  |  |  |  |  |  |
| Independently (reference) | 1 | **--** | **--** | 1 | **--** | **--** |  |  |  |
| Assisted | 0.85 | 0.65-1.10 | 0.220 | 2.32 | 1.14-4.75 | 0.021 | 1.01 | 0.78-1.31 | 0.934 |
| Immobile | 1.09 | 0.77-1.55 | 0.620 | 2.04 | 0.88-4.77 | 0.098 | 0.56 | 0.40-0.80 | 0.001 |
| *Composite of pre-fracture and early mobility* |  |  |  |  |  |  |  |  |  |
| Freely without aids **and** Independently (reference) | 1 | **--** | **--** | 1 | **--** | **--** | **--** | **--** | **--** |
| Outdoors with one aid **or** Assisted | 1.55 | 1.03-2.33 | 0.036 | 1.22 | 0.48-3.11 | 0.683 | 0.68 | 0.47-0.99 | 0.043 |
| Outdoors with two aids/frame or indoors **and** Immobility | 2.33 | 1.36-4.00 | 0.002 | 1.05 | 0.31-3.49 | 0.939 | 0.27 | 0.16-0.47 | <0.001 |

ASA, American Society of Anesthesiologists; AMTS, Abbreviated Mental Test.*Too few pressure ulcers for analysis with composite variables.
